# Effect of health promotion and fluoride varnish on dental caries among Australian Aboriginal children: results from a community-randomized controlled trial*

**DOI:** 10.1111/j.1600-0528.2010.00561.x

**Published:** 2011-02

**Authors:** Gary D Slade, Ross S Bailie, Kaye Roberts-Thomson, Amanda J Leach, Iris Raye, Colin Endean, Bruce Simmons, Peter Morris

**Affiliations:** 1 Department of Dental Ecology, School of Dentistry, University of North Carolina at Chapel HillChapel Hill, NC, USA; 2 Menzies School of Health Research, Charles Darwin UniversityDarwin, NT, Australia; 3 Australian Research Centre for Population Oral Health, School of Dentistry, University of AdelaideAdelaide, SA, Australia; 4 Remote Oral Health Services Consultant, Alice SpringsNT, Australia

**Keywords:** dental caries, fluoride varnish, health promotion, indigenous, randomized controlled trial

## Abstract

**Objectives:**

We tested a dental health program in remote Aboriginal communities of Australia's Northern Territory, hypothesizing that it would reduce dental caries in preschool children.

**Methods:**

In this 2-year, prospective, cluster-randomized, concurrent controlled, open trial of the dental health program compared to no such program, 30 communities were allocated at random to intervention and control groups. All residents aged 18–47 months were invited to participate. Twice per year for 2 years in the 15 intervention communities, fluoride varnish was applied to children's teeth, water consumption and daily tooth cleaning with toothpaste were advocated, dental health was promoted in community settings, and primary health care workers were trained in preventive dental care. Data from dental examinations at baseline and after 2 years were used to compute net dental caries increment per child (d_3_mfs). A multi-level statistical model compared d_3_mfs between intervention and control groups with adjustment for the clustered randomization design; four other models used additional variables for adjustment.

**Results:**

At baseline, 666 children were examined; 543 of them (82%) were re-examined 2 years later. The adjusted d_3_mfs increment was significantly lower in the intervention group compared to the control group by an average of 3.0 surfaces per child (95% CI = 1.2, 4.9), a prevented fraction of 31%. Adjustment for additional variables yielded caries reductions ranging from 2.3 to 3.5 surfaces per child and prevented fractions of 24–36%.

**Conclusions:**

These results corroborate findings from other studies where fluoride varnish was efficacious in preventing dental caries in young children.

Indigenous^1^ Australians experience lamentable rates of death and disease. Life expectancy at birth is 59 years for men and 65 years for women, some 17–18 years below the corresponding figures for non-Indigenous Australians (1). The poor health of Indigenous Australians is attributed to risk behaviors in individuals (e.g. use of tobacco, alcohol and other substances) and to broader societal factors such as the organization of health care and quality of housing. There are less tangible but equally pervasive effects of disempowerment that arise through welfare dependency, loss of traditional roles and feelings of hopelessness (2).

Australia's Indigenous children also experience disproportionately high rates of dental disease. Indeed, during the last 15 years, caries rates have increased among Indigenous Australian children and declined in non-Indigenous children (3). Today's disparities between Indigenous and non-Indigenous Australians are particularly pronounced in the preschool years (4) and in geographically remote areas (5–7). It is noteworthy that these disparities in dental caries are not fully explained by the lower socioeconomic status (SES) of Aboriginal children (6). Higher rates of dental caries in Indigenous children have broader health consequences. In 2002-03, the rate of hospitalization for dental treatment among Aboriginal preschool children was 1.4 times the rate seen in non-Aboriginal preschoolers (8).

When planning this study, we knew that fluoride varnish was effective in reducing levels of decay (9). We also knew that fluoride varnish and parent counseling in diet and oral hygiene could be provided by nondental personnel in primary care settings (10). We saw this as an important prerequisite for the sustainability of any new intervention in remote Aboriginal communities, where routine health care for preschool children is provided by primary health care workers.

To achieve similar benefits in our setting, we felt a preventive dental program should also target families and communities. Researchers attribute disparities in dental caries to behavioral risk factors, such as frequent sugar consumption as well as community-level characteristics, including suboptimal levels of fluoride in drinking water and poor access to dental care (7). Another study cited broader social influences, concluding ‘it could be that factors concerning the social history of Indigenous people contribute more to oral health outcomes than SES per se, which could explain why Indigenous children had worse oral health than non-Indigenous children’ (3).

The views of Indigenous Australians themselves are reported less commonly in the scientific literature. When we consulted with communities prior to starting this project, the ‘old people’ said ‘we never had this problem’ and began sharing stories of traditional health practices and a way of life. We were told some Indigenous Australians still practice a degree of ‘Traditional Medicine’ and they see health as a way of life, encompassing their land, law and culture, spirituality, economic, social, physical, mental and environmental well-being of its people. This was supported by Miriam-Rose Ungunmerr, a respected elder from one of our participating communities, who has described Aboriginal people as ‘Food Gatherers’. She writes ‘These “Food Gatherers”, as well as physical, drew spiritual sustenance from nature and the land. It was this spiritual sustenance that gave them their real strength and the power for such long endurance. They celebrated the land and their closeness to it, even oneness with it, through various ceremonies (11).’

These accounts, together with evidence from the scientific literature, motivated us to develop a program to prevent dental caries that targeted communities, families and children themselves.

## Materials and methods

### Objectives and hypotheses

The goal of the intervention was to prevent dental caries in preschool children living in remote Aboriginal communities in Australia's Northern Territory (NT). The objective of this study is to report the efficacy findings on the primary end point, 2-year net caries increment in primary teeth. We hypothesized that caries increment per child would be lower, on average, in intervention communities than in control communities. Secondary outcomes were measures of dental health behavior and community actions, and will be reported elsewhere.

### Study design

We conducted a 2-year, prospective, cluster-randomized, concurrent controlled, open trial of a dental health program to prevent dental caries compared to no such program. Because of the community component, the randomization design was clustered, meaning that communities were assigned at random to either intervention or control group. All children within any given community were to receive the same study procedures. Children in both intervention and control groups were dentally examined at the time of enrollment and 2 years later to permit calculation of net caries increment, the primary end point used to determine efficacy. The research team had no other involvement in the control communities during the 2-year follow-up period.

### Participants

Inclusion criteria for communities were as follows: (i) remote location (>100 km from Darwin); (ii) classified as Aboriginal (i.e. management by an Indigenous council of community members); (iii) sufficient population (at least 5 births per annum); and (iv) signed, informed consent to participate in the study from the community council. The first three criteria were established through reference to published records, while the fourth criterion was established through a process of community consultation, as described later. Inclusion criteria for children within participating communities were as follows: (i) Aboriginal identity, as declared by parent or family member; (ii) permanent residency in the community, not an outstation, as defined by the council's population list, updated after consultation with community leaders; (iii) age 18 months to less than 48 months; (iv) no reported history of asthma; and (v) signed, informed consent of parent or family member.

### Consultation with and enrollment of communities into the study

The project began in October 2005, with a 9-month period of community consultation to verify eligibility and to guide development of the community-level interventions. The consultation process also fulfilled requirements of the National Health and Medical Research Council and the Indigenous Advisory Committee of the Menzies School of Health Research.

A letter and information sheet describing the study was mailed and faxed to each community council along with follow-up telephone contact to solicit interest in a consultation visit. Communities that provided signed consent for the consultation visit were then visited by study personnel who spoke with council members, community elders and other community leaders in a 1-day visit. Information was provided about dental decay and its prevention, and community members were asked to describe their priorities for dental health of their young children. Information was sought about existing resources relevant to children's health and welfare, including preschool and day-care groups, food sold at community stores, sources of drinking water and health services, including dental services. Community members were asked to nominate aspects of their community that they believed could be strengthened to help prevent dental disease. The information was used to implement a common strategy for advice and health promotion in all intervention communities. Study personnel also explained the research project, including the requirement that participating communities would be allocated to the intervention or control groups at random. The intended benefits and known risks were outlined, as well as the roles of study personnel, children, family groups, health care workers and community groups. Discussions with health care staff focused on their knowledge and practices regarding dental decay, its prevention and treatment. When communities expressed interest in participating, the study personnel sought recommendations for individuals to serve on the study's Indigenous Reference Group (IRG).

### Randomized allocation of communities to experimental groups

Following the consultation phase, consenting communities were allocated to either an intervention group or control group. Prior to randomization, six strata were formed, based on three characteristics of study communities: (i) timing of community consent; (ii) population size; and (iii) geographic region^2^. The first three strata were formed by the 14 communities that consented to participate in the study by March 2006. Stratum 1 comprised four moderate-sized communities in the Top End; Stratum 2 comprised six moderate-sized communities in the Centre; and Stratum 3 comprised small communities in the Centre. The remaining three strata were formed by communities that consented to participate after March 2006: Stratum 4 comprised 10 moderate-sized communities in the Katherine region; Stratum 5 comprised 4 moderate-sized East-Arnhem communities; and Stratum 6 comprised two large communities in the Top End. At the time of randomization, information was incomplete on levels of fluoride in drinking water of participating communities, although historical records showed that naturally occurring fluoride was present in four of the communities in Stratum 2 and in probably in a few of the communities in Strata 3 and 4. Furthermore, we knew that Strata 1, 5 and 6 had negligible amounts of fluoride in drinking water.

Within each stratum, communities were block-allocated at random to achieve equal numbers of intervention and control communities within strata. A random allocation algorithm was created by a consultant statistician using Stata software. He allocated communities from the first three strata after receiving signed consent to participate from each of those communities. Similarly, he allocated the last three strata after all communities in those strata provided signed consent. The list of community allocations was provided to project personnel before they visited communities to recruit children. Because community-level health promotion activities were self-evident, there was no attempt to conceal community allocation, from either children, community groups or study personnel.

### Study interventions

Between May 2006 and December 2008, teams of 2-4 study personnel made five visits to each of the 15 intervention communities. Visits occurred at approximately 6-month intervals and lasted 2–5 days per visit. At each visit, three types of interventions were provided for all eligible children and communities in the intervention group.

- Duraphat^3^ fluoride varnish was applied to children's teeth once every 6 months for 2 years with the aim to complete five applications per child. The first application took place after the baseline dental epidemiological examination, and the final application was administered after the follow-up examination (see below). In almost all instances, varnish was applied by clinical study personnel: dental therapists or dentists. Exceptions occurred when it was applied by health center personnel who were trained in the clinical procedures by the research team.

Using a standardized clinical protocol (12), children were positioned in a knee-to-knee position with a parent or family member helping to hold the child. The teeth were first cleaned with a toothbrush but no toothpaste. Teeth were dried with absorbent paper pads. A single drop of approximately 0.25 ml Duraphat varnish was dispensed, and a thin film was painted onto all visible tooth surfaces using a small foam-tipped brush. Priority was given to maxillary anterior teeth, followed by maxillary molars, then mandibular molars and, finally, mandibular incisors. The intention was to use all 0.25 ml of varnish, but no additional varnish was dispensed if that was insufficient. Excess varnish on the soft tissues was removed with gauze, and the parent or family member was asked to insure that the child abstained from food and drink for the following 30 min.

- Advice to parents and family groups about caries prevention was provided in two settings. The first was during varnish application where the clinician explained the causes of dental decay and methods to prevent it. This included advice about drinking water, limiting sugar exposure, use of fluoride-containing toothpaste and tooth brushing. After demonstrating tooth brushing, each parent/family member was given the toothbrush, a tube of low-concentration fluoride toothpaste^4^ and a children's sized, reusable water bottle. The second setting was children's play groups and preschools, where the same information and products were provided to parents and family members.
- Community health promotion engaged parents, store owners, community leaders and health care workers about oral health and prevention of dental decay in their community. This took place in settings ranging from ‘face painting days’ to formal presentations at community council meetings. In addition to reinforcing information presented to parents and family groups, information was provided about community-wide activities to promote oral health. For example, specific information was provided regarding fluoridation of community drinking water. At community stores, proprietors were encouraged to order supplies of children's toothpaste and toothbrushes that were provided at reduced cost by Colgate-Palmolive Pty Limited. Recognizing that Aboriginal Health Workers are the principal health care providers who promote traditional health practices, we explained the process of tooth decay to them, placing emphasis on the potential caries-preventive benefits of traditional health practices and ‘bush tucker’ (ie. food gathered from the land).

Reinforcement of the same health promotion messages was conveyed to primary health care workers in health centers. Health center staff were trained in oral disease recognition and referral of children with dental decay to school dental services. Training was supported with chart books and DVD instruction. We encouraged health workers to apply fluoride varnish to all teeth and to keep records of such procedures. This meant that any applications to study participants could be identified by project clinicians, reducing potential for more than one application every 6 months. Training was repeated in many communities owing to a high rate of turnover of health center staff.

### Dental examination and referral at intervention and control communities

In intervention and control groups, baseline dental epidemiological examinations were conducted when children enrolled in the study. Follow-up examinations were conducted 2 years later. Examiners advised parents and family members of any dental treatment needed by the child. When there were signs of fever or a spreading dental abscess, immediate dental treatment was recommended. For other children with localized abscesses or caries-related pain, family members were advised to seek dental treatment as soon as possible. For other children with dental caries, the recommendation was for dental care at a time that was convenient.

### Other services operating during the study period

During the study period, community health centers provided routine medical services in both intervention and control communities. In most communities, the centers were staffed by a nurse and/or Aboriginal Health Worker. Periodic visits were made by a general practitioner medical doctor, usually once per week. In remote communities, these primary health care workers are encouraged to follow a documented, standard protocol that outlines steps for managing acute dental infections, primarily using antibiotics and pain control medication^5^. Additionally, the Children's Dental Service of the NT Department of Health and Families provided comprehensive, general dental treatment for school-aged children, including examinations, preventive care, fillings and tooth extraction. At the time of this study, funding of this service was limited and access in remote communities was highly variable.

### Outcome measures

The primary end point to determine efficacy of the intervention was net dental caries increment (d_3_mfs), a child-level measurement. This represents the number of tooth surfaces that became decayed or were treated for dental caries (by filling or extraction) during the 2-year study period. Dental decay was enumerated at the threshold of cavitation, that is, a visible break in the enamel surface caused by caries-demineralization.

### Sources of data

Net dental caries increment, d_3_mfs, was calculated using information from the baseline and 2-year dental epidemiological examinations. Examinations were conducted by eight registered dental therapists hired for the study. Before both examination periods, examiners completed a 2-day training and calibration program where they reviewed the 18-page examination protocol and practiced the examination procedures among preschool children who were not study subjects. During calibration, any uncertainty or disagreement was discussed and resolved by a reference examiner, Dr. Colin Endean, a dentist who had experience in dental examination surveys in Aboriginal communities (5).

Clinicians assessed caries experience of all primary teeth using a battery-illuminated dental mirror but no explorer. Unerupted and missing teeth were noted, and if a tooth could be verified as extracted as a result of caries, all five surfaces were recorded as missing owing to caries. Teeth were dried with absorbent paper, and the status of five surfaces on each primary tooth was evaluated separately. Each surface was classified according to the most severe finding, represented in ascending order of severity as: (i) sound, (ii) opacity with no loss of enamel; (iii) hypoplastic loss of enamel; (iv) precavitated caries with no break of enamel (e.g. white spot); (v) filled owing to dental caries; (vi) arrested, cavitated carious lesion (i.e. hardened enamel or dentine in the base of a cavity); and (vii) cavitated carious lesion (i.e. with break of enamel). If the examiner suspected that caries had arrested in a cavitated lesion, a ball-ended, plastic periodontal probe was used to determine that the lesion felt hard. However, no tactile instruments or criteria were used to distinguish between precavitated and cavitated lesions.

During data collection, inter-examiner reliability was measured between the ‘gold-standard’ dentist and each of the examiners. For each examiner, we aimed to conduct five pairs of replicate examinations. In each pair, a single child was examined once by the study examiner and once by the gold-standard dentist, with neither person knowing the examination findings recorded by the other.

Children's age and sex was recorded during interviews with parents or family group members at the time they provided consent for their child to participate in the study. Study personnel also recorded fluoride varnish application and community health promotion activities. When health center personnel applied varnish, treatment records were audited to count such applications.

Community-level data were obtained from published records. This included population size and distance to the nearest regional hospital as a proxy for remoteness. Data on concentration of fluoride in drinking water were obtained from the Power and Water Corporation of the NT, based on routine sampling of community water supplies conducted over the study period. When communities had more than one source of drinking water, the average concentration was calculated.

### Statistical analysis

Net dental caries increment was calculated by comparing baseline and follow-up data that were paired by child, tooth and surface to enumerate surfaces that had either a caries increment or decrement (13). An increment was defined as a surface that, at baseline, was either unerupted, sound, opaque, hypoplastic or precavitated and that, at follow-up, was either missing as a result of caries, filled, arrested or cavitated. A decrement was defined as a surface that, at baseline, was missing as a result of caries, filled, arrested or cavitated and that, at follow-up, was either sound, opaque, hypoplastic or precavitated. Because cavitation is not reversible, decrements in this study represent errors by examiners and/or recorders, so net caries increment was calculated to correct for such errors (13). Each child's net d_3_mfs increment was computed by summing the number of surfaces with an increment and subtracting the number of surfaces with a decrement.

For the statistical evaluation of intervention efficacy, we conducted a two-tailed test of the null hypothesis that the mean net d_3_mfs caries increment per child was equivalent in intervention and control communities. Consistent with the analytic protocol, we used SAS proc mixed to create a multi-level, linear regression model that generated estimates of efficacy adjusting for the clustered and the stratified study design. Net d_3_mfs caries increment was the dependent variable in the model, treatment allocation was the predictor variable, community was a random-effect intercept, and stratum number was a categorical, fixed effect covariate. We define this as our *a priori* model. It was an intent-to-treat, complete case analysis from all children who had both baseline and 2-year follow-up examinations. The model's least squares means provided adjusted net d_3_mfs increment per child and associated 95% confidence intervals (CIs) for intervention and control groups. The measure of intervention efficacy was the difference between intervention and control groups in adjusted net d_3_mfs increment per child. The 95% CI was the estimate's precision, and if it excluded the null value of zero, the difference between groups was judged to be statistically significant. This efficacy estimate represents the average number of tooth surfaces, per child, in which dental caries was prevented as a result of the intervention - in other words, the average number of cavities prevented, per child. The prevented fraction was also computed, that is, the efficacy estimate divided by adjusted mean net d_3_mfs increment in the control group.

Four additional analytic strategies explored findings that arose from the *a priori* model. (1) Community-level measures of population size, distance to the nearest hospital and concentration of fluoride in drinking water were added as nonrandomized, fixed effect covariates to the *a priori* model and retained if statistically significant (*P* < 0.05). This model sought to further adjust the efficacy estimate for observed differences between study groups in those community characteristics. (2) To provide comparability with other studies evaluating efficacy, children's age, sex and baseline d_3_mfs were added as fixed effect covariates to the *a priori* model. (3) Intent-to-treat analysis among all enrolled children was undertaken by imputing data for net d_3_mfs increment among the 123 children lost to follow-up. Net d_3_mfs increment was regressed against three predictor variables (age, sex and baseline d_3_mfs) for 543 children with complete data. The vector of parameter estimates from that model was then multiplied by age, sex and baseline d_3_mfs of the 123 children with no follow-up examination to impute their net d_3_mfs caries increment. The imputed data were pooled with complete case data, creating data for 666 children that were evaluated using the *a priori* analytic model. (4) Varnish dose-response was investigated by sub-classifying children in the intervention group into four groups according to the number of fluoride varnish applications: 0–3, 4, 5 or 6–8. Those groups were then used instead of the single treatment group as the main predictor in the *a priori* model.

### Inter-examiner reliability

Inter-examiner reliability between examiners and the gold-standard dentist was evaluated on all erupted tooth surfaces of children with replicate examinations. Contingency tables were created between paired surface-specific diagnoses, dichotomized to signify presence of caries experience (missing as a result of caries, filled, arrested or cavitated) or absence of caries experience (sound, opaque, hypoplastic or precavitated). The level of agreement was expressed as the kappa statistic.

### Sample size justification

We planned to enroll 700 children from at least 20 communities after calculating that those numbers would provide sufficient power of 80% to detect a 35% reduction in caries using a two-tailed test of statistical significance and a critical *P*-value of *P* < 0.05. This effect size was within the 95% CI of 19-48% reported for prevented fraction in a meta-analysis of three placebo-controlled trials of fluoride varnish (9). Although our intervention included additional health promotion components that plausibly could have increased the effect size reported for fluoride varnish alone, we were conservative because Aboriginal children in remote communities have high caries rates and very frequent exposure to caries risk factors, all of which could diminish any benefit of the intervention.

Because there were no caries increment data for Aboriginal preschool children, the sample size calculation used surveillance data from 4-year-old NT children where mean dmft was 2.87 and standard deviation was 3.26 (14). We multiplied the standard deviation by square root of 2.0 (3.26 × 1.41 = 4.60) to allow for an expected design effect of 2.0 (i.e. intra-cluster correlation of 0.03) because of clustering of children in communities. The nominated effect size was 35% × 2.87 = 1.0. This yielded a requirement of 325 subjects per group, which we increased to an enrollment target of 350 per group, in anticipation of approximate 5% loss to follow-up based on our experience in conducting other studies in remote NT communities. There were no planned or actual interim analyses. Only one primary end point was to be used, so there was no adjustment of the conventional threshold of *P* < 0.05 for type I error.

### Ethical conduct of research

This project was reviewed and approved by the Health Research Ethics Committees of the Menzies School of Health Research and Department of Health and Families in Darwin, Central Australia, and the University of Adelaide. IRG for the project was set up by the project coordinator, Ms Iris Raye. The IRG met twice a year and provided advice and feedback to the investigators. Community leaders signed an informed consent statement signifying willingness for their community to be in the study. A parent or family group member provided signed informed consent for their child's participation.

## Results

**Fig. 1:**
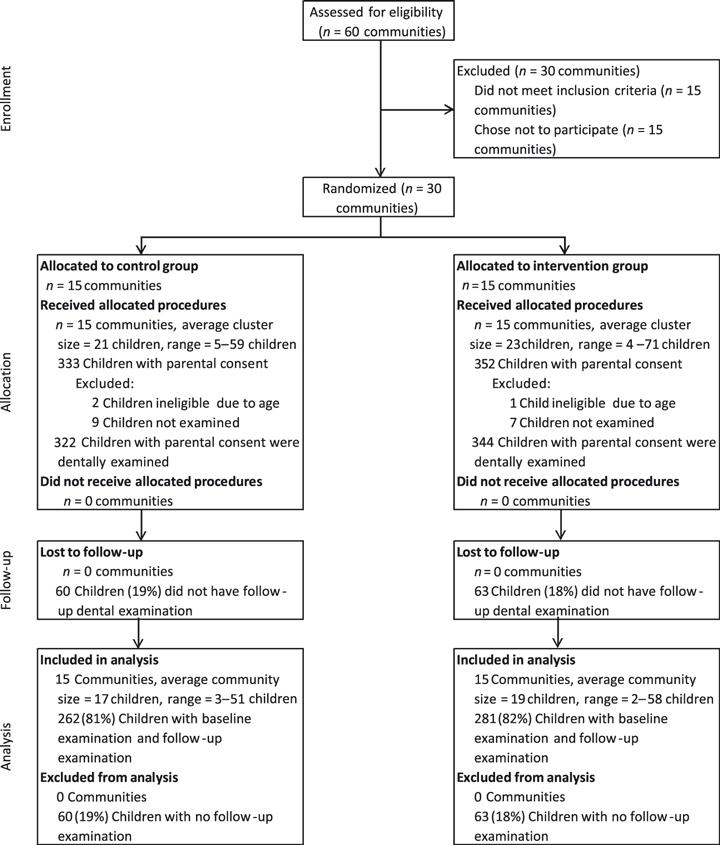
Flowchart of recruitment and follow-up visits.

Of the 60 remote Aboriginal communities in the Northern Territory, 15 were excluded because they were too small or inaccessible. A further 15 communities chose not to participate during the consultation phase (October 2005–June 2006), leaving 30 consenting communities that were randomized (Fig. 1). Parents of 685 children provided consent although three such children were excluded because they were ineligible because of age. A further 16 were excluded because they could not be dentally examined. Baseline examinations of 666 children were conducted between May 2006 and December 2006.

The distribution of three community characteristics differed between the 15 control and 15 intervention communities by up to 14% in absolute percentages (i.e., 87% of control communities were ≥250 km from a hospital compared to 73% of intervention communities - Table 1). Greater differences in community characteristics were observed between children in the two groups: 48% of children in the control group lived in small communities (≤450 population) compared to 30% of children in the intervention group (Table 1). Also, 19% of children in the control group lived in places where drinking water contained ≥0.6 ppm F compared to 8% in the intervention group (*P*< 0.05).

**Table 1:** Community characteristics in intervention and control groups at the time of enrollment

	Control group	Intervention group	*P*-value^a^
*Distribution among 30 communities*			
Number of communities	15	15	
Population size: number (%)			
≤450 people	9/15 (60%)	9/15 (60%)	1.00
>450 people	6/15 (40%)	6/15 (40%)	
Distance to nearest hospital: number (%)			
<250km	2/15 (13%)	4/15 (27%)	0.65
≥250 km	13/15 (87%)	11/15 (73%)	
Fluoride concentration in drinking water: number (%)			
<0.6 ppm F	10/15 (67%)	12/15 (80%)	0.68
≥0.6 ppm F	5/15 (33%)	3/15 (20%)	
*Distribution among 666 children*			
Number of children	322	344	
Population size: number (%)			
≤450 people	155/322 (48%)	104/344 (30%)	<0.01
>450 people	167/322 (52%)	240/344 (70%)	
Distance to nearest hospital: number (%)			
<250 km	28/322 (9%)	46/344 (13%)	0.06
≥250 km	294/322 (91%)	298/344 (87%)	
Fluoride concentration in drinking water: number (%)			
<0.6 ppm F	262/322 (81%)	315/344 (92%)	<0.01
≥0.6 ppm F	60/322 (19%)	29/344 (8%)	

a *P*-values test null hypothesis of equivalence between control and intervention using Fisher's exact test.

In contrast, there was no more than 7% net difference in the distribution of baseline demographic characteristics and clinical dental findings between children in the control and intervention communities (Table 2). Furthermore, the mean age of children differed by only 0.6 months, and mean number of tooth surfaces with caries experience per child at baseline differed by only 0.3 d_3_mfs. Nearly two thirds of children had some caries experience at the baseline examination. Almost all caries was untreated: teeth had been extracted in only 10 children, and only one child had fillings (Table 2).

**Table 2:** Children's characteristics in intervention and control groups at the time of enrollment

	Control group	Intervention group	*P*-value*
Number of children	322	344	
Age: number of children (%)			
18–<30 months	117/322 (36%)	124/344 (36%)	0.25
30–<40 months	119/322 (37%)	110/344 (32%)	
40–<48 months	86/322 (27%)	110/344 (32%)	
Age in months: mean (95% CI)	33.0 (32.1, 33.9)	33.6 (32.7,34.5)	0.31
Sex: number of children (%)			
Male	169/322 (52%)	171/344 (50%)	0.47
Female	153/322 (48%)	173/344 (50%)	
Caries experience: number of children (%)			
No cavitated carious surfaces	117/322 (36%)	134/344 (39%)	0.52
One or more cavitated carious surfaces	205/322 (64%)	210/344 (61%)	
No arrested carious surfaces	297/322 (92%)	302/344 (88%)	0.07
One or more arrested carious surfaces	25/322 (8%)	42/344 (12%)	
No filled surfaces	322/322 (100%)	343/344 (100%)	1.00
One or more filled surfaces	0/322 (0%)	1/344 (0%)	
No missing tooth surfaces	318/322 (99%)	338/344 (98%)	0.75
One or more missing tooth surfaces	4/322 (1%)	6/344 (2%)	
No caries experience (d_3_mfs^a^ = 0)	113/322 (35%)	125/344 (36%)	0.53
One or more surfaces with caries experience (d_3_mfs > 0)	209/322 (65%)	219/344 (64%)	
Number of surfaces with caries experience per child [d_3_mfs]: mean (95% CI)	4.6 (3.9, 5.2)	4.9 (4.2, 5.6)	0.55
Other dental conditions: number of children (%)			
No precavitated carious surfaces	161/322 (50%)	181/344 (53%)	0.61
One or more precavitated carious surfaces	161/322 (50%)	163/344 (47%)	
No surfaces with opacity	96/322 (30%)	109/344 (32%)	0.52
One or more surfaces with opacity	226/322 (70%)	235/344 (68%)	
No surfaces with hypoplasia	209/322 (65%)	214/344 (62%)	0.75
One or more surfaces with hypoplasia	113/322 (35%)	130/344 (38%)	
Recommendation for caries treatment: number of children (%)			
No caries treatment needed	188/322 (58%)	175/344 (51%)	0.06
Treatment needed when convenient	121/322 (38%)	144/344 (42%)	
Treatment needed as soon as possible	13/322 (4%)	25/344 (7%)	
Treatment needed immediately	0/322 (0%)	0/344 (0%)	

a The d_3_mfs index is each child's number of cavitated, arrested, filled or missing tooth surfaces.

* *P*-values test null hypothesis of equivalence between control and intervention groups using Chi-square test (age groups, treatment recommendation), t-tests (mean age, mean d_3_mfs) or Fisher's exact test (all other comparisons)

All 30 communities adhered to allocated study procedures, including follow-up examinations (Fig. 1). However, 123 children (18% of 666) did not receive a follow-up examination. Usually, this was because the child had left the community or could not be located during the research team's final visit. Rates of follow-up differed by no more than 5% in absolute percentage between subgroups of children classified according to community characteristics, age, sex and baseline caries experience (Table 3).

**Table 3:** Variation in rate of loss to follow-up between baseline and 2-year examinations

	Number (%) of children lost to follow-up	*P*-value*
All children	123/666 (18%)	
*Community factors*		
Study group		
Control communities	60/322 (19%)	0.92
Intervention communities	63/281 (18%)	
Population size		
≤450 people	51/259 (20%)	0.54
>450 people	72/407 (18%)	
Distance to nearest hospital		
<250 km	16/74 (22%)	0.43
≥250 km	107/592 (18%)	
Fluoride concentration in drinking water		
<0.6 ppm F	105/577 (18%)	0.66
≥0.6 ppm F	18/89 (20%)	
*Child factors*		
Age		
18–<30 months	51/241 (21%)	0.23
30–<40 months	43/229 (19%)	
40–<48 months	29/196 (15%)	
Sex		
Male	57/326 (17%)	0.55
Female	66/340 (19%)	
Baseline d_3_mfs		
0 surfaces	50/238 (21%)	0.40
1–5 surfaces	40/223 (18%)	
≥6 surfaces	33/205 (16%)	

* *P*-values test null hypothesis of equivalence in % followed up between rows using Chi-square test (age group, baseline d_3_mfs) or Fisher's exact test (all other comparisons)

In the intervention group, 450 community health promotion activities were provided, ranging from 14 to 101 per community. The most frequent activities were training health care workers in oral screening and varnish application (76 activities), meetings with community groups (58 activities) and work with community stores (54 activities). Children in the intervention group received 1,207 fluoride varnish applications, ranging from 0 (1 child) to 8 applications (one child): 271 children (96% of 281) received between two and six applications. The median was 5 applications per child. Study personnel administered 1,190 varnish applications, and the remaining 17 were performed by health center personnel.

Examiner reliability in diagnosing caries experience used data from 13 children at baseline, yielding paired examination findings for 1,252 tooth surfaces. Kappa for reliability of all four baseline examiners compared to the gold-standard dentist was 0.80 (95% CI = 0.72, 0.87). For individual examiners compared to the gold standard, kappa ranged from 0.73 (0.54, 0.92) to 0.83 (0.71, 0.94). At follow-up examinations, all four examiners were assessed for reliability among 21 children, yielding paired examination findings for 2070 tooth surfaces. Kappa for reliability of all four examiners at follow-up compared to the gold-standard dentist was 0.83 (95% CI = 0.80, 0.87). For individual examiners compared to the gold standard, kappa ranged from 0.68 (0.58, 0.78) to 0.90 (0.84, 0.95).

At the follow-up examination, 94% of children (510/543) had caries experience, and most of the caries was untreated: only 7% of children (37/543) had filled teeth, 3% (19/543) had extracted teeth, and 6% had both filled and extracted teeth. Examiners noted that most children (475/543 = 87%) needed treatment for decay, including 17% of children (95/543) who needed care urgently. One or more dental abscesses were noted in 78 children at the follow-up examination.

In the 2-year interval, caries increment was computed for 4858 tooth surfaces, while caries decrement was computed for 45 surfaces among the 543 children who were re-examined after 2 years. The net caries increment of 4813 tooth surfaces in the cohort equated to an average net d_3_mfs increment of 8.9 new carious surfaces per child during the 2-year period (range = −4 to +60 surfaces per child, median = 6 surfaces per child, interquartile range = 2 - 12 surfaces per child). A total of 89% of children (484/543) had a net caries increment of one or more d_3_mfs surfaces during the 2 years between baseline and follow-up examinations. The percentage was identical in both intervention and control groups. The average period between baseline and follow-up examinations was 24 months (range = 20–29 months), and 90% of follow-up examinations were conducted 23–25 months after the baseline examination.

The adjusted net d_3_mfs increment was statistically significantly lower among children in the intervention group compared to the control group by an average of 3.0 surfaces per child (95% CI = 1.2, 4.9) based on the *a priori* analysis that adjusted for clustering of children within communities and the stratified sampling design (Table 4). The prevented fraction for the adjusted estimates was 31%, signifying that nearly one third fewer carious lesions per child developed in the intervention group than in the control group.

**Table 4:** Net 2-year d_3_mfs caries increment and estimated effects of intervention on children's net 2-year d_3_mfs caries increment

		Extension of *a priori* model adjusting for			
		
	*a priori* model (1)	Community factors (2)	Child factors (3)	Loss to followup (4)	Varnish dose response (5)
Number of communities/number of children in analysis					
Control group	15/262	15/262	15/262	15/322	15/262
Intervention group	15/281	15/281	15/281	15/344	15/281
Unadjusted net d_3_mfs increment per child: mean (95% CI)					
Control group	10.1 (8.9, 11.4)	10.1 (8.9, 11.4)	10.1 (8.9, 11.4)	9.8 (8.8, 10.1)	10.1 (8.9, 11.4)
Intervention group	7.7 (6.8, 8.5)	7.7 (6.8, 8.5)	7.7 (6.8, 8.5)	8.0 (7.2, 8.7)	7.7 (6.8, 8.5)
Adjusted net d_3_mfs increment per child: mean (95% CI)					
Control group	9.9 (8.5, 11.3)	9.7 (8.5, 10.9)	9.4 (8.0, 10.8)	9.6 (8.5, 10.7)	9.9 (8.5, 11.3)
Intervention group	6.9 (5.5, 8.2)	6.2 (5.0, 7.4)	7.0 (5.6, 8.3)	7.3 (6.2, 8.4)	
0–3 varnish applications versus control					7.1 (4.4, 9.8)
4 varnish applications versus control					6.2 (4.2, 8.2)
5 varnish applications versus control					7.1 (5.3, 8.9)
6–8 varnish applications versus control					8.6 (3.7, 13.5)
Effect estimates: difference in adjusted net d_3_mfs increment per child: mean (95% CI)					
Efficacy of intervention versus control group	−3.0 (−4.9, −1.2)	−3.5 (−5.1, −1.9)	−2.4 (−4.3, −0.6)	−2.3 (−3.7, −0.8)	
Effect of additional 1ppm F		−4.3 (−7.0, −1.6)			
Effect of age (years)			−0.3 (−0.3, −0.2)		
Effect of baseline d_3_mfs (no. of surfaces)			0.5 (0.4, 0.6)		
Prevented fraction	31%	36%	26%	24%	
Intra-cluster correlation coefficient	0.02	0.00	0.02	0.01	0.02

(1) *A priori* model is complete case, intent-to-treat analysis using multi-level linear regression model adjusted for fixed effect of stratum and random effect of communities.

(2) Addition to *a priori* model of fluoride concentration in drinking water as fixed effect covariate.

(3) Addition to *a priori* model of child's age and baseline d_3_mfs as fixed effect covariates.

(4) *A priori* model applied to 666 subjects by adding regression-imputation values of d_3_mfs increment for 123 children lost to follow-up. This produced an imputed intent-to-treat analysis of all randomized subjects.

(5) Replacement of binary study group from *a priori* model with dummy variables for control group and four categories of fluoride varnish applications.

The adjusted efficacy estimate increased, in absolute value, to −3.5 surfaces per child (95% CI = −5.1, −1.9, prevented fraction = 36%) when the *a priori* model was extended to additionally adjust for fluoride concentration in drinking water (Table 4). Because their effects were found to be statistically nonsignificant, population size (*P* = 0.19) and distance to nearest hospital (*P* = 0.96) were excluded from the model. In this model, an additional 1 ppm F in drinking water was associated with a reduction in d_3_mfs increment of 4.3 surfaces per child (95%CI = 1.6, 7.0). In contrast, when age and sex were added to the *a priori* model, the adjusted efficacy estimate decreased, in absolute value, to −2.4 surfaces per child (95% CI = −4.3, −0.6; prevented fraction = 26%). When data were imputed for net d_3_mfs increment among children lost to follow-up, the analytic method used in the *a priori* model yielded an adjusted efficacy estimate of −2.3 (95% CI = −3.7, −0.8; prevented fraction = 24%). There was no evidence of varnish ‘dose response’ when the *a priori* model was altered to estimate effects of four categories of fluoride varnish application compared the control group (Table 4).

There were no protocol deviations and no adverse events detected during the study.

## Discussion

This preventive dental program of twice-yearly fluoride varnish application combined with community health promotion significantly reduced the average number of tooth surfaces, per child, that developed caries in a 2-year period compared to the level observed in control communities. Depending on the analytic assumptions, the intervention reduced net d_3_mfs caries increment by 2.3-3.5 surfaces, per child. This represented 24–36% (respectively) fewer tooth surfaces per child that developed dental caries over 2 years. These reductions occurred despite dental caries being virtually ubiquitous among these children.

It is plausible that the estimated efficacy measures should range from 2.3 to 3.5 fewer decayed surfaces, per child, given the different factors that were investigated in the first four analytic models. We believe that the *a priori* model underestimates the true efficacy of this intervention, because children in intervention communities were less likely to be exposed to fluoride in drinking water than children in control communities. In principle, random allocation should have prevented this imbalance. The probability of such imbalance is a hazard of clustered randomized trials, when the number of clusters is relatively small (*n* = 30 communities in this study). Ideally, we could have reduced the probability of this imbalance by first stratifying communities based on fluoride concentration in drinking water. However, we did not have complete information about fluoride levels when communities were enrolled and allocated. Instead, we used region and population size as proxy indicators that we expected would optimize the probability of equivalence in baseline characteristics between study groups.

The analytic model that adjusted for fluoride in drinking water therefore represents our best effort at post hoc correction of this imbalance between community groups. Also of note was the finding that fluoride in drinking water had an effect that was statistically significant and independent of the intervention. In fact, an increase of 1 ppm F in drinking water was associated with an average reduction of 4.3 carious surfaces, per child. Although that is an observed association, not a finding from a randomized treatment allocation, the implication is that a nonfluoridated community that adopted this intervention and increased concentration of fluoride in its water supply to 1 ppm F could expect an average reduction of 3.5 + 4.3 = 7.8 fewer carious surfaces, per child – more than halving the caries rate.

In contrast, the efficacy estimate reduced when the *a priori* model was further adjusted for child's age, sex and baseline caries. We report this finding solely for comparability with other studies, but we believe the result is biased through over-adjustment (15), because there was similar distribution of these characteristics between the study groups and none of the characteristics was associated with variation in loss to follow-up. Imputing data using regression-based methods necessarily biases efficacy estimates toward the null, so it is not surprising that the fourth model yielded the smallest efficacy estimate of 2.3 fewer carious surfaces, on average, per child. However, this sensitivity analysis illustrates the most conservative estimate of effect (given that we could not measure caries increment in 123 children).

There was a larger loss of subjects than we anticipated before the study, when we calculated the required sample size. Conversely, though, our predicted design effect used when calculating sample size was unnecessarily conservative - in fact, intra-cluster correlation for communities was 2% or less for each of the models, and average number of children per cluster was 22, so the true design effect was no greater than 1.4. The greater power owing to the small design effect therefore provided a trade-off against the reduced power associated with loss to follow-up of 123 children.

For all analytic models, net effects of the intervention are within the range reported in a systematic review of fluoride varnish, where the pooled estimate of prevented fraction in primary teeth was 33% (95% CI = 19%, 48%).(9) Also, our results are similar to findings from a study reported since that systematic review. In a 2-year, community-randomized trial among Canadian Aboriginal children aged 6 months–5 years, fluoride varnish and caregiver counseling reduced caries increment by an average of 2.8 surfaces per child, a prevented fraction of 18% (16).

In contrast, the net efficacy effects in this Australian study were considerably greater than the net reduction of 1.0 surface per child reported in a 2-year individually randomized clinical trial of fluoride varnish among children aged 6–44 months from low-income San Francisco families. However, our prevented fraction of 24–36% was considerably lower than the prevented fraction of 61% observed in that US study (17). This apparent paradox merely reflects properties of two different effect measures: net differences in caries increment are an absolute measure of effect, whereas prevented fractions are an index of relative effects. Absolute and relative effects are both valid measures, although they answer different questions about health (18). The former signify the typical effect that can be expected for individuals who receive the intervention, while the latter are informative for population health by estimating the fraction of disease in a population that can be prevented by the intervention. In this Australian setting, the absolute reduction in caries, *per child*, was greater than that in the San Francisco study, but because caries was virtually ubiquitous among these Australian children, a smaller fraction of the disease was prevented compared to children studied in San Francisco.

A further consequence of the near ubiquity of dental caries was a lack of measurable difference between study groups in the proportion of children developing one or more carious lesions. The proportion, which represents the conventional measures of cumulative disease incidence, was 89% in both intervention and control communities. Because there was zero net difference between groups in cumulative incidence, the conventional calculation of number needed to treat was undefined. The implication is that not even a single child would be protected *completely* from caries onset because of this intervention, even if it was provided to an infinite number of children. In contrast, in the Canadian study, 75% of children in control communities and 71% in intervention communities developed caries, so the number needed to treat was 26 (16). Arguably, for this setting, a more realistic measure of therapeutic benefit than number needed to treat *to prevent caries completely* would be the number needed to treat to prevent 100 cavities: that is, 100 divided by the efficacy estimate. Results from our *a priori* model indicate that 100÷3.0 = 33 children would need to receive the intervention to prevent 100 cavities.

Aside from technical properties of effect measures, our study differs from others noted previously in that our intervention included significant work providing community-based health promotion. Scientifically, it would have been preferable to use a factorial study design with four allocation groups to assess independent and joint effects of varnish versus community health promotion. Findings from such a study design would provide more valid comparison with studies and systematic reviews where the intervention has been limited to fluoride varnish. However, there were insufficient communities to undertake the larger study that would be required for a factorial design. This limitation also hampers the investigation into ‘dose response’, because we have no measure of ‘dose’ for community health promotion. This may be one reason that there was no observable difference in caries increment across four levels of varnish application studied in the ‘dose response’ analytic model. Another potential bias in the assessment of dose response is the fact that children were not allocated to different frequencies of varnish application. In fact, it is likely that children who received fewer or more applications than intended differed systematically from children who received the five intended applications.

We probably would have achieved larger caries reductions if children used toothpaste with a greater concentration of fluoride than recommended in our intervention. We considered promoting adult-strength toothpaste, rather than ‘My First Colgate’ that contains 0.45 mg/g of fluoride, but we deferred toward the latter because it was the standard of care for young children in Australia when we began the study. However, soon after we began the intervention, new Australian guidelines were published, advocating use of adult-strength toothpaste, containing around 1 mg/g of fluoride, for young children at high risk of caries (19). We were unwilling to change the study protocol because of the potential for introducing bias. However, given the evidence that adult-strength fluoride toothpaste would be beneficial for high-risk children, we now encourage implementation of that recommendation in any new programs.

Regrettably, for the children in this study, only a small percentage of decayed teeth was treated. This is despite the fact that some form of dental treatment was recommended for the majority of children following baseline dental examinations. Usually, though, such treatment would have entailed a long trip to a regional center to receive care, so it is not surprising that there was little evidence of dental treatment. However, from the perspective of measurement validity, it meant there was little potential for inflation of the dmfs index owing to treatment decisions unrelated to caries, a documented shortcoming of the index (20).

In fact, the general shortage of dental treatment services for preschool children in these remote communities underscores the urgency of a preventive program to help reduce the burden of dental disease. It was therefore disappointing that so few varnish applications were provided by primary health care workers in community health centers. In principle, they are the best people to sustain these preventive dental services because they see preschool children sufficiently frequently to permit repeated applications of varnish. Other studies have shown that it is feasible to train nurses and other primary health care workers in these dental preventive procedures for them to incorporate the procedures into medical practice (10).

For those reasons, we devoted considerable effort to training primary health care workers with the intention that they would provide many of the preventive dental services planned for this study. Our efforts failed for several reasons. Primary health care workers face heavy demands in providing medical care for this underserved population. Another barrier was the high turnover of remote health staff in these communities, resulting in new staff unfamiliar with the training provided. It was also conceivable that our presence created an impression that it would be redundant for primary health care workers to provide dental preventive procedures. It is to be hoped that the success of this project in reducing caries will add impetus to policies recommending active involvement of primary health care workers in an ongoing dental prevention program.

While these findings corroborate evidence from diverse settings where fluoride varnish is efficacious in preventing dental caries in young children, it is clear that the varnish, even when coupled with community health promotion, does not eliminate the problem. In fact, the intervention itself prevented no more than one quarter to one third of new cavities. And although fluoride in drinking water was associated with prevention of as many cavities again, it was striking that, even in intervention communities, 89% of children developed caries during the 2-year period. Given the pervasive effects on ill health of factors such as disempowerment through welfare policy, it would be naive to believe that these Australian Aboriginal children could be ‘immunized’ against caries, even with the combination of this preventive program and fluoride in drinking water. This limitation is not unique to dental disease. When commenting on interventions to improve general health, Paradies and Cunningham praised even piecemeal gains, arguing to ‘redefine a large problem into a series of smaller, more manageable problems, and to aim for ‘small wins’ that eventually add up (2).

During the consultation process, we learned there is potential for interventions that build on strengths within communities, such as traditional medicine and bush tucker. Because we had limited resources to actively support communities in those traditions, we instead encouraged Aboriginal Health Workers to include the ‘tooth story’ in their promotion of traditional health practices. One consequence was that the remaining components of the intervention had a noticeably European approach to caries control: fluoride varnish, water consumption and daily tooth cleaning with toothpaste. Given that caries levels remained high, even in intervention communities, we believe additional dental health benefits could be obtained by investing more resources in promoting traditional health practices.

The imperative now is to implement what is known to work. Based on these results, we recommend that local health staff in remote Aboriginal communities receive training and support in the delivery of a comprehensive program to prevent dental caries. Further studies are needed to identify additional interventions that may reduce the burden of disease even further. More resources should be allocated to identifying healthy initiatives already promoted by Aboriginal families.

## Footnotes

1 Indigenous Australians comprise Aboriginals and Torres-Strait Islanders. This study was limited to Aboriginal children living in remote Aboriginal communities in the Northern Territory of Australia. This paper uses both collective terms ‘Indigenous’ and ‘Aboriginal’ because individuals, organizations and publications relevant to the study use both terms.

2 The Top End is the most northern area of the Northern Territory, and it has a tropical climate; the Centre is the area around Alice Springs, and it has a desert climate; Katherine is the area south of Darwin surrounding the town of Katherine, and it has a sub-tropical climate; and East Arnhem is in the north east of the Territory, and it has a tropical climate.

3 5% sodium fluoride (2.26 % fluoride ion). Colgate-Palmolive Pty Ltd, Level 15, 345 George St, Sydney, NSW 2000, Australia.

4 ‘My First Colgate’, 0.324 sodium monofluorophosphate. Colgate-Palmolive Pty Ltd, Level 15, 345 George St, Sydney, NSW 2000, Australia.

5 Cited at http://www.carpa.org.au/manual_reference.htm on 12 October, 2009
